# Adiposity and blood pressure among 55 000 relatively lean rural adults in southwest of China

**DOI:** 10.1038/jhh.2014.129

**Published:** 2015-02-05

**Authors:** X Chen, H Du, J Zhang, X Chen, G Luo, X Que, N Zhang, Z Bian, Y Guo, L Li, Z Chen, X Wu

**Affiliations:** 1 West China School of Public Health, Sichuan University, Sichuan Province, China; 2 Sichuan Provincial Center of Disease Prevention and Control, Sichuan Province, China; 3 Clinical Trial Service Unit and Epidemiological Studies Unit (CTSU), Nuffield Department of Population Health, University of Oxford, Oxford, UK; 4 Pengzhou Center of Disease Prevention and Control, Sichuan Province, China; 5 Chinese Academy of Medical Sciences, Beijing, China; 6 School of Public Health, Peking University, Beijing, China

## Abstract

Obesity is a strong determinant of blood pressure. Uncertainty remains, however, about which indices of adiposity most strongly predict blood pressure, particularly among those who were relatively lean, such as those from rural China. We analyzed cross-sectional data on 55 687 (38.3% men) participants aged 30–79 years who were enrolled into the China Kadoorie Biobank from a rural county in southwest of China during 2004–2008. Measured body mass index (BMI) and waist circumference (WC) were related to blood pressure in multivariable linear regression analyses. The overall mean values of BMI, WC, systolic blood pressure (SBP) and diastolic blood pressure (DBP) were 23.3 kg m^−2^, 78.0 cm, 129.2 mm Hg and 77.2 mm Hg, respectively. There was a strongly positive, and apparently linear, relationship of BMI and WC with blood pressure, with 1 s.d. higher BMI associated with 4.3/2.3 mm Hg higher SBP/DBP and 1 s.d. WC associated with 3.8/2.1 mm Hg (*P*<0.0001). Additional adjustment for WC only slightly attenuated the association of BMI with blood pressure, whereas additional adjustment for BMI almost completely eliminated the association of WC with blood pressure. Our findings suggest that in relatively lean Chinese adults, general adiposity is more strongly assciated with blood pressure than central adiposity.

## Introduction

High blood pressure is a major cause of cardiovascular disease morbidity and mortality in both developed and developing countries. One of the major determinants of blood pressure is adiposity,^1, 2, 3^ which could be broadly divided into general adiposity that is commonly measured by body mass index (BMI) and central adiposity with waist circumference (WC) as the most common measure.^4, 5^ Despite generally consistent findings of positive association between adiposity and blood pressure from studies in different populations, questions remain, however, as to which measure of adiposity is more strongly associated with blood pressure,^6, 7, 8, 9, 10^ whether the strengths of associations differ importantly between populations, and whether the associations are modified significantly by age, gender and lifestyle factors (for example, smoking, alcohol consumption, physical activity and diet). A good understanding of these questions can inform the assessment of potential impacts of the recent obesity epidemic on blood pressure and related vascular burden in different populations.

Several studies have investigated the associations of different measures of adiposity with blood pressure, but findings have been inconsistent. Most of them tended to suggest that WC is either similarly or more strongly associated with blood pressure as compared to BMI.^6, 7, 8^ But a few others did not support this, showing that BMI was a better predictor of blood pressure than WC.^9, 10^ A large majority of these previous studies were conducted among Western populations where most adults are overweight or obese and individuals with hypertension are generally well managed. In China, however, hypertension is highly prevalent and is still poorly detected and managed.^11^ Moreover, despite the recent increase, prevalence of overweight or obesity remains low, especially in rural China.^12, 13^ Reliable assessment of the associations between different adiposity measures and blood pressure in relatively lean Chinese population would not only help to improve our understanding about possible mechanisms linking adiposity with blood pressure, but also inform the development of effective public health policies for control of hypertension and related cardiovascular diseases.

To help fill this knowledge gap, we report a cross-sectional analysis of data from over 55 000 men and women from rural China who were enrolled into the nationwide China Kadoorie Biobank study.^14^ The main objectives of the present study are (1) to determine, both qualitatively and quantitatively, the associations of general and central adiposity with blood pressure as well as the prevalence of hypertension; and (2) to assess whether the associations differ by age, gender and lifestyle factors.

## Materials and methods

### Study population

Participants were all from Pengzhou, a rural county in the south of Sichuan Province, China, who were enrolled during 2004–2008 as a part of the China Kadoorie Biobank. Details about the design, conduct and data collection of the China Kadoorie Biobank have been described previously.^14, 15^ Briefly, it involved 10 geographically defined localities (5 rural and 5 urban) across China chosen to cover wide spectra of risk exposures and disease patterns. All adults who were permanent residents, aged 35–74 years, in defined regions with no major disability were invited to participate in the study. A total of 55 687 (38.3% men, overall response rate 26.0%) participants were enrolled from Pengzhou, Sichuan.

The study was approved by the ethics committees of the University of Oxford, the Chinese Center for Disease Control and Prevention, and the Sichuan Center for Disease Control and Prevention. Written informed consent was obtained from all participants.

### Data collection

The baseline survey took place in temporary study clinics, specially set up near participants’ residential areas. All the data collection was undertaken by trained research staff according to standard protocols and procedures.^14, 15^ An interviewer-administered computerized questionnaire was used to collect information on sociodemographic characteristics, lifestyle factors (that is, diet, physical activity, smoking and alcohol consumption), medical history and so on.

A range of physical measurements were also undertaken. Height was measured to the nearest 0.1 cm using a portable stadiometer when participants wearing no hat and shoes. Weight was measured using bioelectrical impedance (TANITA-TBF-300GS: Tanita Corp., Tokyo, Japan) while participants wearing no shoes and only light clothes. BMI was calculated as weight in kilograms divided by the square of height in meters (kg m^−2^). WC was determined to the nearest 0.1 cm using a soft non-stretchable tape at midway between the lowest rib and the iliac crest. Blood pressure was measured twice using a UA-779 digital monitor recommended by the British Hypertension Society (A & D Instruments Ltd., Abingdon, UK). If the systolic blood pressure (SBP) difference between the first two measurements was more than 10 mm Hg, a third measurement was obtained and the last two measurements were recorded in the database. For the current analysis, we used mean of the two blood pressure measurements. Hypertension was defined as either prior physician-diagnosed hypertension, having measured mean SBP⩾140 mm Hg, or diastolic blood pressure (DBP) ⩾90 mm Hg.

### Statistical methods

All analyses were performed first separately for men and women and then in overall population. Multiple linear regression models (with a statement of LSMEANS) were used to estimate the mean values of BMI and WC by sociodemographic and lifestyle factors and season of survey, with adjustment, where appropriate, for age (in 5-year intervals), education (three groups) and annual household income (three groups). Prevalence rates of overweight (BMI⩾25 kg m^−2^) and central obesity (WC⩾90 cm for men or ⩾80 cm for women)^16^ in each of these subgroups were calculated with standardization for the same covariates as mentioned above using logistic regression.

Multiple linear regression models were also used to estimate the least square means of blood pressure, and logistic regression models were used to calculate the adjusted prevalence of hypertension by BMI and WC, adjusting for age, education, income, smoking status, alcohol and fresh fruit consumption, sedentary leisure time and season of survey. For BMI, participants were categorized into seven categories, with cut points 18.5, 21, 24, 25, 28 and 30 kg m^−2^. This categorization incorporated current definitions of underweight, normal weight, overweight and obesity defined by both WHO and the Working Group on Obesity in China.^17^ Likewise, seven categories were used for WC, with cut points of 70, 75, 80, 85, 90 and 95 cm. The 95% confidence interval (CI) of hypertension prevalence was estimated using the ‘floating absolute risk’ method.^18^ For the analyses on blood pressure, 2109 participants who were using antihypertensive treatment at the time of survey were excluded, leaving 53 578 in those analyses.

Finally, differences (and their 95% CIs) in blood pressure per 5 kg m^−2^ BMI were calculated both overall and in subgroups by sex, age, education, income, smoking status, alcohol and fresh fruit consumption, sedentary leisure time and seasons of survey, using multiple linear regression models with adjustment for above mentioned covariates.

In addition, analyses were also carried out to examine the associations of other general (that is, percentage of body fat) and central (that is, waist-to-hip ratio (WHR) and waist-to-height ratio) adiposity indices with blood pressure.

All these analyses were performed using SAS 9.2 (SAS Institute Inc., Cary, NC, USA) and R version 2.10.1 (R Foundation for Statistical Computing, Vienna, Austria) was used to graph results.

## Results

Of the study participants, the overall mean (s.d.) age was 51.0 (10.5) years, and means (s.d.) of SBP and DBP were 129.2 mm Hg (19.0) and 77.2 mm Hg (10.2), respectively. About a quarter of our participants were hypertensive, slightly more in men than in women. On average, men were 2 years older, had 3.8/2.6 mm Hg higher SBP/DBP, and were more likely to smoke (67.1 vs 9.5%) and drink regularly (50.4 vs 6.2%) compared with women (Table 1).

**Figure 1:**
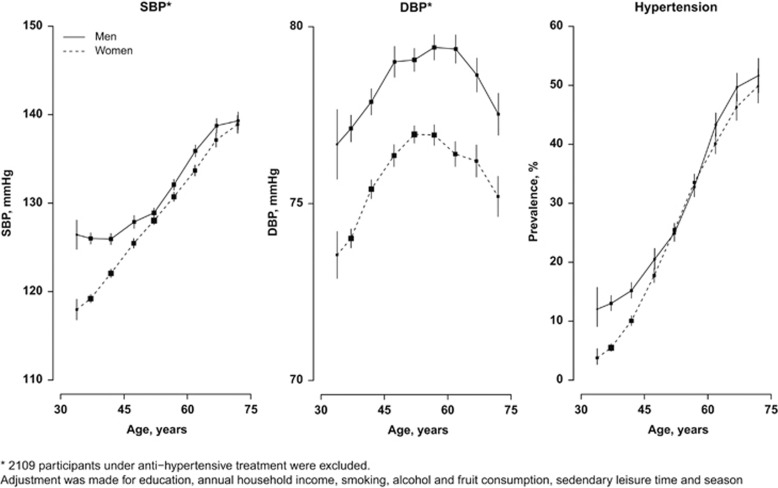
Associations of systolic blood pressure (SBP), diastolic blood pressure (DBP) and prevalence of hypertension with age. The means of blood pressure were calculated for each age group (5-year interval), adjusting for education, annual household income, smoking, alcohol and fruit consumption, sedentary leisure time and season.

The mean BMI was 22.9 kg m^−2^ in men and 23.6 kg m^−2^ in women, and the mean WC was 78.9 and 77.4 cm, respectively. In men, more than three quarters (77.4%) had BMI<25 kg m^−2^, with only 1.5% being obese (BMI⩾30 kg m^−2^). In women, the corresponding rates were 68.5 and 3.8%, respectively (Tables 2 and 3). According to WC, 87.8% men and 62.3% women had no central obesity. With the exception of the youngest groups (30–39 years), mean BMI tended to decrease with increasing age in both men and women, but no similar trend was seen for WC. SBP showed a strongly positive relationship with age (Figure 1), with the mean SBP being 12.9 mm Hg (men) and 20.9 mm Hg (women) higher at age 70 years or above than at age 30–35 years. By contrast, no linear trend with age was seen for DBP, which increased first for about 3 mm Hg from 30–35 to 55–60 years and then decreased gradually afterwards. Prevalence of hypertension rose steadily with increasing age, with the absolute differences between youngest and oldest age groups being 39.7 and 46.1% in men and women, respectively.

In men, those with higher education level or household income had higher BMI and WC (Table 2). However in women, although higher household income was associated with a higher adiposity, those with higher education tended to be less obese (Table 3). Compared with never/non-regular smokers, current regular smokers have lower but ex-smokers had higher levels of adiposity. A higher level of adiposity was found in ex-regular alcohol drinkers, those who consumed more fresh fruits, and those who spent more time in sedentary activities. There were also small variations in mean BMI and WC across different seasons, with those enrolled in summer having the lowest BMI but highest WC, and those in winter having the highest BMI but lowest WC (Tables 2 and 3).

**Figure 2:**
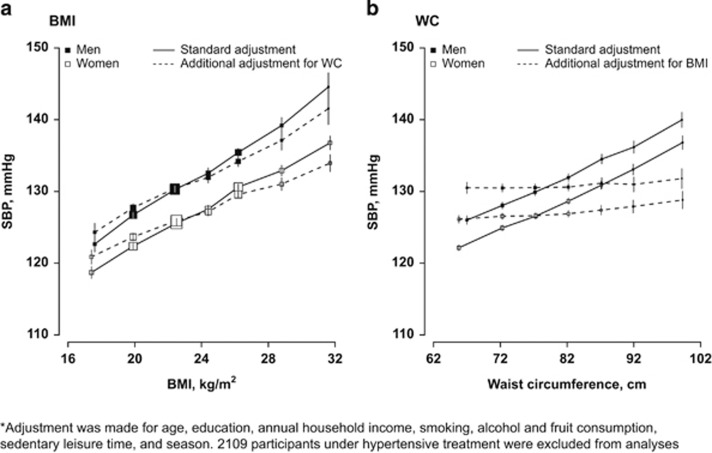
Systolic blood pressure (SBP) in relation to body mass index (BMI), **a** and waist circumference (WC), **b** among 53 578 participants. The means of SBP were calculated for each BMI and WC group, with adjustment for age, education, annual household income, smoking, alcohol and fruit consumption, sedentary leisure time, season and with/without the adjustment for each other.

As shown in Figure 2, both BMI and WC were strongly and positively associated with SBP, before adjusting for each other, with 1 s.d. higher BMI (3.2 kg m^−2^) associated with 4.3 mm Hg (95% CI: 4.2–4.4) and 1 s.d. WC (8.9 cm) associated with 3.8 mm Hg (3.6–3.9). After the additional adjustment for WC, the associations of BMI with SBP were only slightly attenuated to 3.8 mm Hg (3.5–4.0). However, the additional adjustment for BMI almost completely eliminated the associations between WC and blood pressure, down to 0.6 mm Hg (0.3–0.9; *P*<0.001). Similar was observed for DBP: additional adjustment for WC only slightly attenuated the associations of BMI with DBP from 2.3 to 1.8 mm Hg for per s.d. BMI, while additional adjustment for BMI dramatically reduced the association of WC with DBP from 2.1 to 0.6 mm Hg per s.d. WC (Supplementary Figure 1).

**Figure 3:**
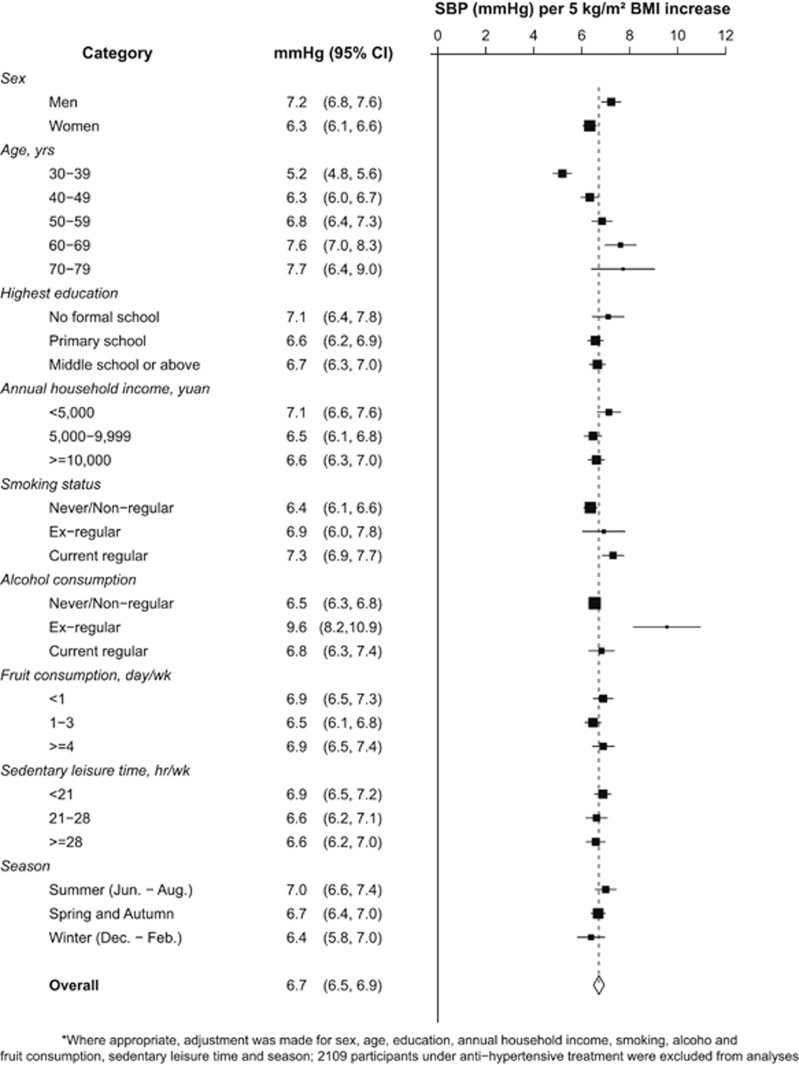
Increment of systolic blood pressure for each 5 kg m^−2^ BMI increase in different subgroups (*n*=53 578)*. SBP differences were calculated with adjustment for age, education, annual household income, smoking, alcohol and fruit consumption, sedentary leisure time and season.

After adjusting for potential confounders, each 5 kg m^−2^ BMI higher was on average associated with 6.7 mm Hg (95% CI: 6.5, 6.9) higher SBP (7.2 mm Hg in men and 6.3 mm Hg in women), with the association being stronger in older than in younger people (5.2 mm Hg at 30–39 years vs 7.7 mm Hg at age 70 or above; Figure 3). The association was also much stronger in ex-regular alcohol drinkers. As shown in Supplementary Figure 2, every 5 kg m^−2^ higher BMI was associated with 3.6 (3.5, 3.7) mm Hg DBP, with the association being stronger in men and in ex-regular drinkers. But no age trend was seen.

Prevalence of hypertension was positively associated with BMI and the association was only slightly attenuated after the additional adjustment for WC (Supplementary Figure 3). Among those in the highest BMI group (BMI⩾30 kg m^−2^), 57.6% of men and 41.1% of women were hypertensive, more than three times as high as those in the lowest BMI group. For WC, additional adjustment for BMI largely reduced its positive association with hypertension (Supplementary Figure 3), with the absolute difference in the prevalence of hypertension between the highest and lowest WC group changed from 37.4–14.5% in men and from 34.4–13.8% in women.

Additional analyses using percentage of body fat to replace BMI (Supplementary Figure 4) and using WHR (Supplementary Figure 5) or waist-to-height ratio (Supplementary Figure 6) to replace WC provided essentially same results.

## Discussion

In this large study of over 55 000 rural adults from China who were relatively lean compared with western populations, the positive association of general adiposity with blood pressure, indicated by BMI or percentage of body fat, was largely independent of central adiposity, with each 5 kg m^−2^ higher BMI associated with 6.7/3.6 mm Hg higher SBP/DBP. Central adiposity as indicated by WC, WHR or waist-to-height ratio, however, was not strongly associated blood pressure after additionally adjusting for general adiposity.

The current study has a rather large sample size, which enabled us to analyze with sufficient precision the independent association of blood pressure with BMI and WC, which are highly correlated with each other (Spearman's correlation coefficient 0.84). In addition, use of carefully measured anthropometrics and blood pressure is another major strength of our study. Self-reported adiposity measures, as used in many previous studies, are subject to reporting bias that may distort the associations differently depending on the levels of adiposity.^19, 20^ The relatively low participation rate may be a limitation of the current study. However, our study population was not intended to be representative of the general population in China or in Sichuan Province.^21, 22, 23^ Moreover, a relatively low response rate will neither bias the observed association nor significantly reduce the generalizability of the associations observed.^21, 22, 23, 24^ Although we were not able to compare the characteristics of those participated and those who did not, the large number of participants and the fact that the observed associations differed very little across various subgroups of individuals are supportive of the study findings being generalizable to the Chinese population at large. Another potential limitation of our current study is its cross-sectional nature. In spite of this, the causal link between adiposity and blood pressure can still be inferred with confidence based on published literatures.

Despite previous research, it is still under debate as to which measurement of adiposity could better predict the risk of cardio-metabolic diseases, such as hypertension, diabetes and heart disease and mortality.^25, 26^ Our study findings contrast with a number of cross-sectional studies previously conducted in either Asian or Western populations, in which WC was shown either more closely or similarly related to blood pressure or risk of hypertension as compared to BMI.^10, 27, 28^ In a study of 14 924 people from the third US National Health and Nutrition Examination Survey (NHANES III) during 1988–1994 in which the mean BMI was 25.4 kg m^−2^, WC was found as an independent predictor of hypertension after adjusting for BMI, with each 1 cm larger WC associated with 3 and 5% higher prevalence of hypertension in men and women, respectively. However, BMI was no longer a predictor of hypertension after adjusting for WC.^27^ In another two studies of Asian^28^ and non-smoking Greek adults participating in the EPIC (European Prospective Investigation into Cancer and Nutrition) study,^10^ however, BMI and WC were found both significantly associated with blood pressure after mutually adjusted for each other.

Several small prospective studies have reported similar adiposity—blood pressure associations as we observed in the present study.^2, 29^ In a study of 202 young US adults with 6-month follow-up, baseline and change of BMI were both significantly associated with change in blood pressure even after the adjustment for baseline and change in WHR. However, association of baseline or change in WHR with blood pressure did not persist after additional adjustment for BMI.^29^ In another study of 3312 Japanese adults with 2-year follow-up, change in BMI was shown to be significantly associated with blood pressure difference after controlling for baseline and change in WC, whereas converse was not true.^2^

The exact cause of the non-significant association between WC and blood pressure after adjusting for BMI is unknown, as are the mechanisms linking adiposity and blood pressure. It has been speculated that increased body volume caused by excessive body fat could lead to a higher peripheral vascular resistance and a series of dysfunction, such as adiponectin deficiency, hyperleptinemia, elevated renin-angiotensin-aldosterone system hormones and mineralocorticoid-stimulating factors.^30, 31, 32^ These dysfunctions may in turn activate the renin-angiotensin-aldosterone system and sympathetic nervous system, which lead to endothelial dysfunction, impaired pressure natriuresis and vascular hypertrophy, and eventually raise blood pressure. As compared to WC, BMI has been found more strongly associated with total body fat mass and body volume.^33, 34^ Therefore, it might be more closely related to peripheral resistance and aforementioned dysfunctions.

The stronger association of BMI with blood pressure per five units higher BMI in men than in women could be explained by the larger s.d. of BMI in women: five BMI units represented 1.7 s.d. in men but only 1.5 s.d. in women. When calculated using per 1 s.d. BMI for SBP, there was no difference in the strength of the association between men and women (4.2 mm Hg in both). The causes for the extremely stronger BMI—blood pressure association in those ex-regular drinkers have yet to be fully understood. It might be true that ex-drinkers might have stopped drinking due to diseases or pre-disease conditions, such as arthrosclerosis, which could modify the association of adiposity with blood pressure. Also, they might have distinct characteristics in lifestyle factors other than the ones we have considered and adjusted for. Elevated arterial wall thickening and stiffening with age may be the potential element leading to a stronger association of BMI with SBP in older people.^35^ Moreover, the stronger association in older people may also reflect the vascular damage of adiposity cumulated during a longer time period. This finding may also implicate a necessity of body weight control among older people for the sake of preventing severe cardiovascular events. The seemingly unexpected positive association between fruit consumption and adiposity might be due to confounding by other dietary factors and/or socioeconomic status. Further investigation in this aspect is outside of the scope of the current paper.

To conclude, our results provide strong evidence that general adiposity, indicated by BMI, was more strongly associated with blood pressure than WC in Chinese adults. A greater emphasis should be placed on BMI for the purpose of monitoring hypertension risk in Chinese adults.
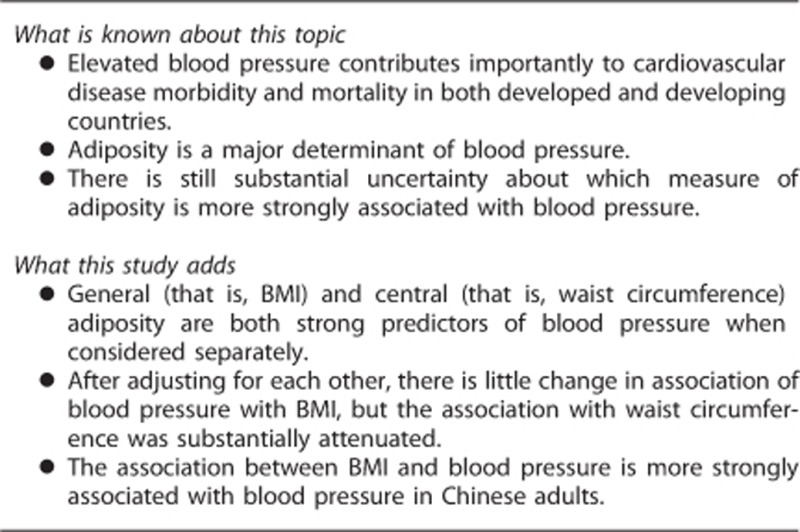


## Figures and Tables

**Table 1:** Main characteristics of study population

*Characteristics*	*Men (*n=*21 315)*	*Women (*n=*34 372)*	*Overall (*n=*55 687)*
*Age, years*			
30–39	15.3	18.8	17.5
40–49	24.3	28.6	26.9
50–59	32.4	32.5	32.5
60–69	21.6	16.1	18.2
70–79	6.4	4.1	5.0
Mean (s.d.)	*52.3 (10.8)*	50.2 (10.3)	51.0 (10.5)
			
*Highest education*			
No formal school	9.6	19.1	15.4
Primary school	52.6	48.2	49.9
Middle school or above	37.9	32.8	34.7
			
*Annual household income, yuan*			
<5000	26.9	27.4	27.2
5000–9999	33.1	36.7	35.4
⩾10 000	40.0	35.8	37.4
			
*Smoking status*			
Never/non-regular	21.5	86.5	61.6
Ex-regular	11.4	3.9	6.8
Current regular	67.1	9.5	31.6
			
*Alcohol intake*			
Never/non-regular	43.1	91.8	73.2
Ex-regular	6.6	1.9	3.7
Current weekly	50.4	6.2	23.1
			
*Fresh fruit consumption, days week^−1^*			
<1	39.8	32.5	35.3
1–3	42.1	43.8	43.1
⩾4	18.1	23.7	21.5
			
*Sedentary leisure time, h week^−1^*			
⩽21	45.9	46.2	46.1
21–28	23.3	25.9	24.9
>28	30.8	28.0	29.1
Mean (s.d.)	*26.7 (11.2)*	*26.0 (10.2)*	*26.3 (10.6)*
			
*SBP, mm Hg*			
<120	23.2	34.0	29.9
120–139	51.5	45.6	47.8
⩾140	25.3	20.4	22.3
Mean (s.d.)	*131.6 (18.4)*	*127.8 (19.3)*	*129.2 (19.0)*
			
*DBP, mm Hg*			
Mean (s.d.)	*78.8 (10.3)*	*76.2 (10.0)*	*77.2 (10.2)*
			
*Hypertension*			
Total^a^	28.7	23.0	25.2
Physician diagnosed	6.4	6.1	6.2
Antihypertensive treatment^b^	57.8	61.0	59.8

Abbreviations: DBP, diastolic blood pressure; SBP, systolic blood pressure.

Values are percentage or otherwise indicated.

a Hypertension was defined as either prior physician diagnosed hypertension, SBP⩾140 mm Hg or DBP⩾90 mm Hg.

b Among those with physician diagnosed hypertension. Italic values indicate mean (s.d).

**Table 2:** BMI, WC and prevalence rates of overweight and abdominal obesity with sociodemographic and lifestyle factors in 21 315 men

	*BMI (kg m^−^*^*2*^)^a^	*BMI*⩾*25 kg m^−^*^*2*^ *(%)*	*WC (cm)*^a^	*WC*⩾*90* cm *(%)*
Overall	22.9 (2.9)	22.6	78.9 (8.8)	12.2
				
*Age, years*				
30–39	22.9 (3.0)	22.3	78.9 (9.0)	12.2
40–49	23.1 (3.0)	23.7	79.3 (9.1)	13.2
50–59	22.9 (2.9)	23.2	78.6 (8.8)	10.9
60–69	22.7 (3.0)	22.7	79.0 (9.0)	13.1
70–79	22.1 (3.0)	16.6	78.4 (9.1)	12.2
				
*Highest education*				
No formal	22.5 (3.1)	18.4	77.7 (9.3)	9.8
Primary school	22.8 (3.0)	21.6	78.7 (9.0)	11.8
Middle school or above	23.0 (3.2)	25.2	79.5 (9.6)	13.3
				
*Annual household income, Yuan*				
<5000	22.4 (3.0)	16.9	77.5 (9.2)	8.8
5000–9999	22.7 (2.9)	20.9	78.2 (8.7)	10.3
⩾10 000	23.3 (3.0)	28.0	80.4 (9.0)	16.1
				
*Smoking status*				
Never/non-regular	23.3 (2.9)	27.5	79.8 (8.7)	14.5
Ex-regular	23.6 (2.9)	32.6	81.1 (8.7)	18.0
Current regular	22.6 (2.9)	19.4	78.2 (8.6)	10.5
				
*Alcohol intake*				
Never/non-regular	22.9 (2.9)^b^	23.4	78.6 (8.7)	12.3
Ex-regular	22.9 (2.9)	24.5	79.2 (8.8)	14.2
Current regular	22.8 (2.9)	21.7	79.1 (8.7)	11.9
				
*Fresh fruit consumption, days week^−1^*				
<1	22.7 (2.9)	20.8	78.5 (8.8)	11.0
1–3	22.9 (2.9)	22.8	78.8 (8.7)	12.4
⩾4	23.2 (2.9)	26.5	80.0 (8.9)	14.5
				
*Sedentary leisure time, h week^−1^*				
⩽21	22.7 (2.9)	19.8	78.0 (8.6)	9.5
21–28	22.8 (2.9)	21.9	78.9 (8.6)	12.0
>28	23.2 (2.9)	27.4	80.2 (8.7)	16.4
				
*Season of survey*				
Summer (June–August)	22.5 (2.9)	20.1	78.9 (8.7)^b^	12.2
Spring and Autumn	22.9 (2.9)	23.3	79.0 (8.7)	12.6
Winter (December–February)	23.1 (2.9)	24.0	78.5 (8.6)	10.8

Abbreviations: BMI, body mass index; WC, waist circumference.

a Values are adjusted mean (s.d.); s.d.=s.e. × sqrt (*n*), where s.e. is the standard error for the adjusted mean and *n* is the number of participants in that group (the rough *n* could be derived from Table 1). Analyses were adjusted for age, education and annual household income, where appropriate.

b *P* for trend >0.05, all other *P* for trend <0.05.

**Table 3:** Associations of BMI, WC and prevalence rates of overweight and abdominal obesity with sociodemographic and lifestyle factors in 34 372 women

	*BMI (kg m^−2^)*^a^	*BMI*⩾*25 kg m^−2^ (%)*	*WC (cm)*^a^	*WC*⩾*80* cm *(%)*
Overall	23.6 (3.3)	31.5	77.4 (9.2)	37.7
				
*Age, years*				
30–39	23.0 (3.4)	23.2	74.7 (9.4)	23.2
40–49	24.0 (3.5)	35.4	77.5 (9.7)	37.4
50–59	23.8 (3.4)	34.8	78.6 (9.3)	43.2
60–69	23.2 (3.5)	29.9	78.3 (9.6)	43.5
70–79	22.3 (3.5)	22.0	77.0 (9.6)	39.1
				
*Highest education*				
No formal school	23.6 (3.7)	31.9	77.5 (10.0)	38.0
Primary school	23.7 (3.4)	33.5	78.0 (9.2)	40.1
Middle school or above	23.3 (3.7)	28.3	76.6 (10.1)	34.0
				
*Annual household income, Yuan*				
<5000	23.4 (3.5)	29.2	77.0 (9.5)	35.7
5000–9999	23.5 (3.3)	31.2	77.3 (9.1)	37.1
⩾10 000	23.7 (3.4)	33.5	77.9 (9.2)	39.7
				
*Smoking status*				
Never/non-regular	23.7 (3.3)	32.3	77.6 (9.1)	38.1
Ex-regular	23.8 (3.4)	34.0	78.6 (9.3)	43.5
Current regular	22.7 (3.4)	22.8	75.7 (9.3)	31.1
				
*Alcohol intake*				
Never/non-regular	23.6 (3.3)	31.6	77.5 (9.0)^b^	37.6
Ex-regular	23.6 (3.3)	33.7	77.9 (9.1)	41.5
Current regular	23.3 (3.3)	28.6	77.5 (9.1)	37.2
				
*Fresh fruit consumption, days week^−1^*				
<1	23.3 (3.4)	29.4	76.8 (9.2)	35.4
1–3	23.6 (3.3)	31.6	77.5 (9.0)	37.9
⩾4	23.8 (3.4)	34.0	78.1 (9.3)	40.4
				
*Sedentary leisure time, h week^−1^*				
⩽21	23.3 (3.3)	28.9	76.8 (9.1)	34.7
21–28	23.7 (3.3)	33.1	77.9 (9.0)	39.5
>28	23.8 (3.3)	34.1	78.2 (9.1)	41.0
				
*Season of survey*				
Summer (June–August)	23.4 (3.3)	29.7	77.9 (9.1)	39.5
Spring and Autumn	23.6 (3.3)	32.2	77.4 (9.0)	37.2
Winter (December–February)	23.6 (3.3)	31.7	76.9 (9.0)	36.3

Abbreviations: BMI, body mass index; WC, waist circumference.

a Values are adjusted mean (s.d.); s.d.=s.e. × sqrt (*n*), where s.e. is the standard error for the adjusted mean and *n* is the number of participants in that group (the rough *n* could be derived from Table 1). Analyses were adjusted for age, education and annual household income, where appropriate.

b *P* for trend = 0.7 and all other *P* for trend <0.05.
