# Erratum: Adiposity and blood pressure among 55 000 relatively lean rural adults in southwest of China

**DOI:** 10.1038/jhh.2017.13

**Published:** 2017-06-07

**Authors:** X Chen, H Du, J Zhang, X Chen, G Luo, X Que, N Zhang, Z Bian, Y Guo, L Li, Z Chen, X Wu

**Keywords:** Hypertension, Body mass index, Prognosis, Epidemiology

**Correction to:** *Journal of Human Hypertension* (2015) **29**, 522–529; doi:10.1038/jhh.2014.129; published online 5 February 2015

This article was originally published under a CC BY-NC-ND 4.0 license, but has now been made available under a CC BY 4.0 license. The PDF and HTML versions of the paper have been modified accordingly.

