# Supplementary material for: Adiposity and blood pressure among 55 000 relatively lean rural adults in southwest of China
Source: J Hum Hypertens. 2015 Feb 5;29(9):522–9. doi: 10.1038/jhh.2014.129 (PMC4537884; doi:10.1038/jhh.2014.129)
Supplement: Supplementary Figure 1 [file jhh2014129x1.pdf]

eFigure 1. Diastolic blood pressure (DBP) in relation to body mass index (BMI) and waist circumference (WC) among 53 578 participants*

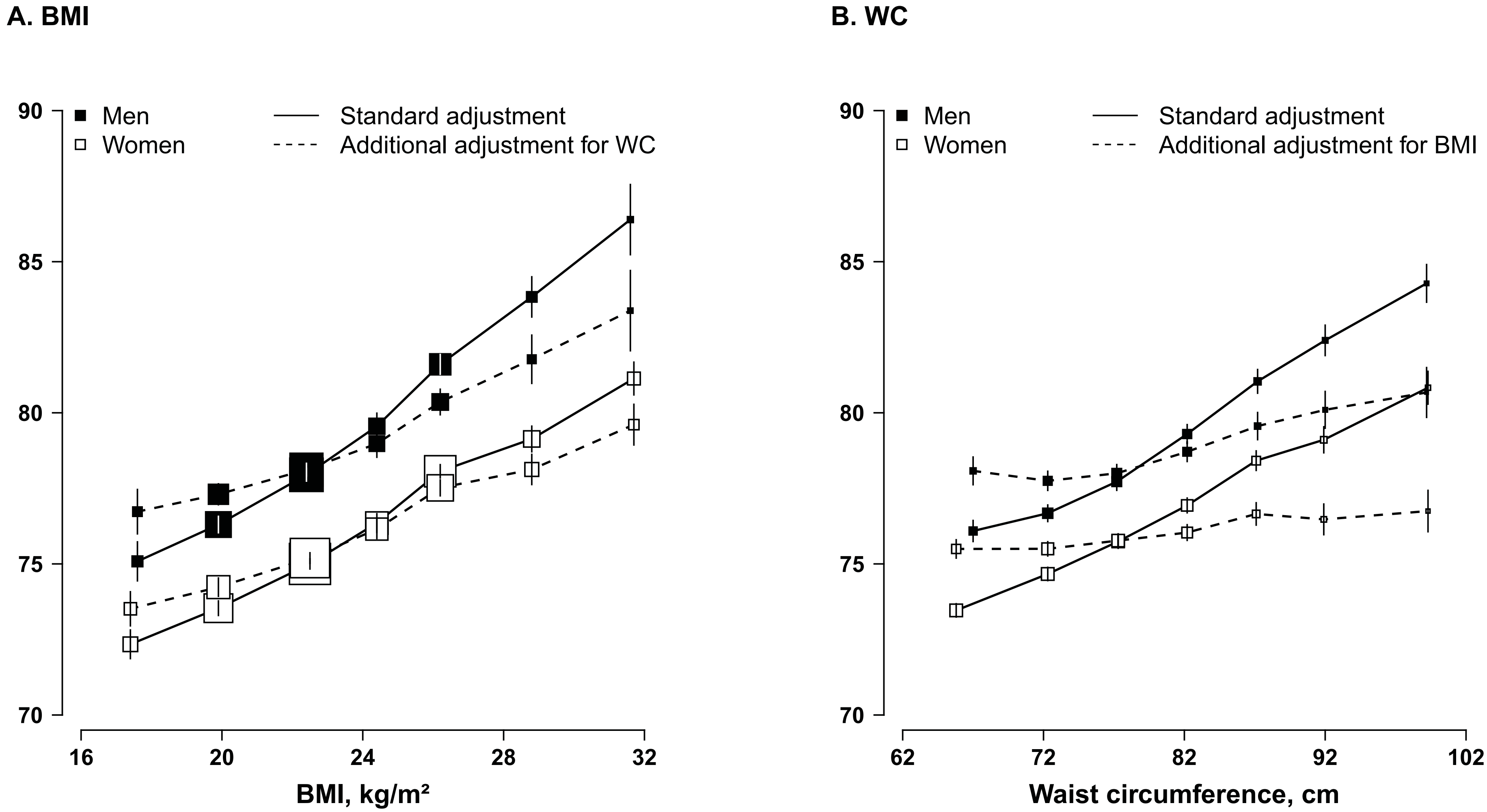

*Adjustment was made for age, education, annual household income, smoking, alcohol and fruit consumption, sedentary leisure time, and season. 2109 participants under hypertensive treatment were excluded from analyses
