# Supplementary material for: Adiposity and blood pressure among 55 000 relatively lean rural adults in southwest of China
Source: J Hum Hypertens. 2015 Feb 5;29(9):522–9. doi: 10.1038/jhh.2014.129 (PMC4537884; doi:10.1038/jhh.2014.129)
Supplement: Supplementary Figure 2 [file jhh2014129x2.pdf]

eFigure 2. Increment of diastolic blood pressure (DBP) for each 5 kg/m² BMI increase in different subgroups (n = 53 578)*

Category	mmHg (95% CI)
Sex	
Men	4.1 (3.9, 4.4)
Women	3.3 (3.2, 3.5)
Age, yrs	
30−39	3.3 (3.0, 3.6)
40−49	3.8 (3.5, 4.0)
50−59	3.7 (3.4, 3.9)
60−69	3.7 (3.3, 4.0)
70−79	3.2 (2.5, 3.9)
Highest education	
No formal school	3.3 (2.9, 3.6)
Primary school	3.5 (3.4, 3.7)
Middle school or above	3.9 (3.7, 4.1)
Annual household income, yuan	
<5,000	3.5 (3.3, 3.8)
5,000−9,999	3.4 (3.2, 3.7)
>=10,000	3.8 (3.6, 4.0)
Smoking status	
Never/Non−regular	3.5 (3.4, 3.7)
Ex−regular	3.1 (2.6, 3.6)
Current regular	3.9 (3.7, 4.2)
Alcohol consumption	
Never/Non−regular	3.5 (3.4, 3.6)
Ex−regular	4.8 (4.0, 5.6)
Current regular	3.8 (3.5, 4.1)
Fruit consumption, day/wk	
<1	3.6 (3.3, 3.8)
01−ÈýÔÂ	3.5 (3.3, 3.7)
>=4	4.0 (3.7, 4.2)
Sedentary leisure time, hr/wk	
<21	3.6 (3.4, 3.8)
21−28	3.6 (3.3, 3.9)
>=28	3.6 (3.4, 3.9)
Season	
Summer (Jun. − Aug.)	3.6 (3.3, 3.8)
Spring and Autumn	3.6 (3.5, 3.8)
Winter (Dec. − Feb.)	3.6 (3.3, 4.0)
**Overall**	3.6 (3.5, 3.7)

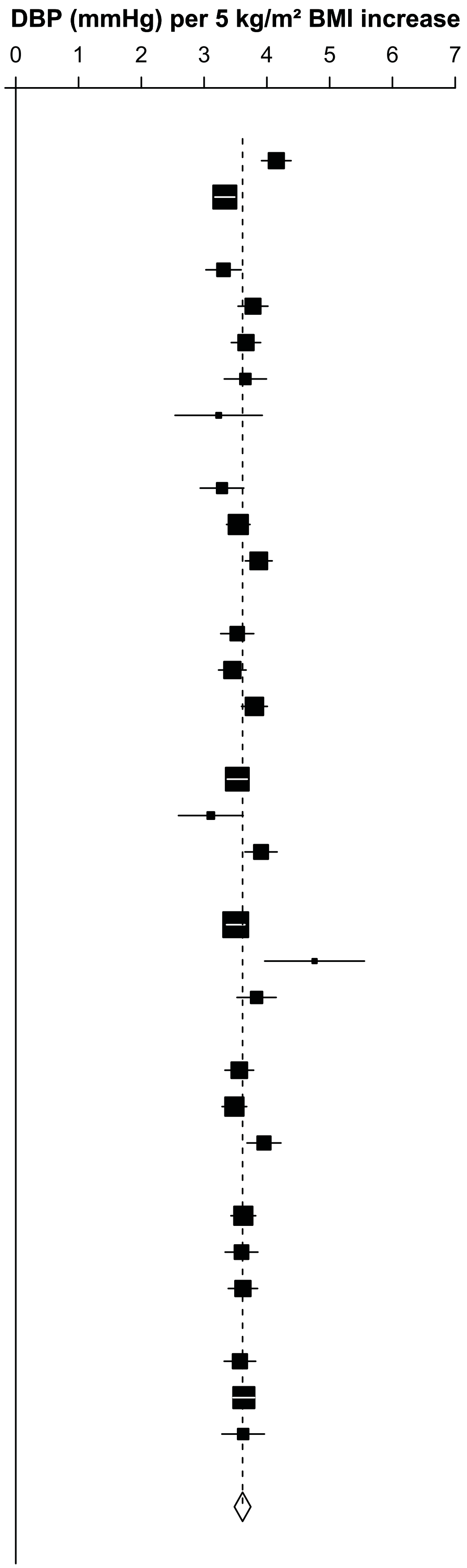

*Where appropriate, adjustment was made for sex, age, education, annual household income, smoking, alcoho and fruit consumption, sedentary leisure time and season; 2109 participants under anti−hypertensive treatment were excluded from analyses
